# Absence of chronic traumatic encephalopathy in retired football players with multiple concussions and neurological symptomatology

**DOI:** 10.3389/fnhum.2013.00222

**Published:** 2013-05-24

**Authors:** Lili-Naz Hazrati, Maria C. Tartaglia, Phedias Diamandis, Karen D. Davis, Robin E. Green, Richard Wennberg, Janice C. Wong, Leo Ezerins, Charles H. Tator

**Affiliations:** ^1^Department of Laboratory Medicine and Pathobiology, University of TorontoToronto, ON, Canada; ^2^Tanz Centre for Research in Neurodegenerative Diseases, University of TorontoToronto, ON, Canada; ^3^Division of Neurology, Krembil Neuroscience Centre, University Health Network, University of TorontoToronto, ON, Canada; ^4^Division of Neurosurgery, Krembil Neuroscience Centre, University Health Network, University of TorontoToronto, ON, Canada; ^5^Division of Brain, Imaging, and Behaviour – Systems Neuroscience, Toronto Western Research Institute, University Health NetworkToronto, ON, Canada; ^6^Department of Surgery, University of TorontoToronto, ON, Canada; ^7^Institute of Medical Science, University of TorontoToronto, ON, Canada; ^8^Research, Cognitive Neurorehabilitation Sciences Lab, Toronto Rehabilitation Institute, University of TorontoToronto, ON, Canada; ^9^Executive Director, Canadian Football League Alumni Association, Members of the Canadian Sports Concussion Project at the Krembil Neuroscience Centre, Toronto Western Hospital and University of TorontoToronto, ON, Canada

**Keywords:** chronic traumatic encephalopathy, repetitive brain injury, professional athletes, dementia, neurodegenerative disease

## Abstract

**Background:** Chronic traumatic encephalopathy (CTE) is the term coined for the neurodegenerative disease often suspected in athletes with histories of repeated concussion and progressive dementia. Histologically, CTE is defined as a tauopathy with a distribution of tau-positive neurofibrillary tangles (NFTs) that is distinct from other tauopathies, and usually shows an absence of beta-amyloid deposits, in contrast to Alzheimer's disease (AD). Although the connection between repeated concussions and CTE-type neurodegeneration has been recently proposed, this causal relationship has not yet been firmly established. Also, the prevalence of CTE among athletes with multiple concussions is unknown.

**Methods:** We performed a consecutive case series brain autopsy study on six retired professional football players from the Canadian Football League (CFL) with histories of multiple concussions and significant neurological decline.

**Results:** All participants had progressive neurocognitive decline prior to death; however, only 3 cases had post-mortem neuropathological findings consistent with CTE. The other 3 participants had pathological diagnoses of AD, amyotrophic lateral sclerosis (ALS), and Parkinson's disease (PD). Moreover, the CTE cases showed co-morbid pathology of cancer, vascular disease, and AD.

**Discussion:** Our case studies highlight that not all athletes with history of repeated concussions and neurological symptomology present neuropathological changes of CTE. These preliminary findings support the need for further research into the link between concussion and CTE as well as the need to expand the research to other possible causes of taupathy in athletes. They point to a critical need for prospective studies with good sampling methods to allow us to understand the relationship between multiple concussions and the development of CTE.

## Introduction

Sport-related concussions affect millions of people in North America annually (Pickett et al., 2004). Among Canadian university hockey players, concussion accounts for 13% of all injures, ranking as the second most common injury after sprains or strains (Rishiraj et al., 2009). Although concussions were previously considered reversible injuries with transient symptoms, a number of recent studies have emerged linking repeated concussions and possibly asymptomatic subconcussive impacts with long-term neurodegenerative changes (McKee et al., 2009, 2010; Omalu et al., 2011; Stern et al., 2011).

The clinical neurocognitive impact of repetitive head injury was described by Martland in 1928 in “poorly skilled boxers” who withstood multiple blows to the head in efforts to position themselves in close proximity to their opponents to land a punch; subsequent symptoms included ataxia, amnesia, dementia, dysarthria, parkinsonism, and other motor and coordination deficits (Martland, 1928; Parker, 1934). In addition, behavioral and personality changes, aggression, jealousy, paranoia, and an increased incidence of physical domestic disputes and suicide were noted (Parker, 1934; Mendez, 1995; McKee et al., 2009; Omalu et al., 2010a).

Recently, the term chronic traumatic encephalopathy (CTE) was coined to refer to the clinical constellation of neurocognitive decline in conjunction with neuropathological findings of abnormal hyperphosphorylated-tau neuronal deposits in a pattern distinguishable from other tauopathies. CTE has been associated with many contact sports, including football, wrestling, hockey, and rugby (Corsellis et al., 1973; Omalu et al., 2005, 2006, 2010a,b,c, 2011; McKee et al., 2009, 2010; Gavett et al., 2011; Stern et al., 2011).

Because of the clinical and social ramifications of CTE and its putative relationship to concussion history, further research is critically needed to better understand the prevalence and risk factors for CTE. As well, the relationship between multiple concussions, positive clinical symptoms and the presence of CTE is also unclear. The current case series examines this relationship.

CTE can only be definitively diagnosed post-mortem. Given the relative rarity of cases of CTE, and the even rarer opportunity for post-mortem examination of individuals with a history of concussions at risk for CTE, it is important to provide a description of these clinical cases as they emerge.

On gross neuropathological examination, typical advanced CTE findings include dilated ventricles, fenestrated cavum septi pellucidi, and significant atrophy of the medial temporal lobes, thalamus, and mammillary bodies (McKee et al., 2009; Gavett et al., 2011; Stern et al., 2011). There can also be pallor of the locus coeruleus and substantia nigra (McKee et al., 2009; Gavett et al., 2011; Stern et al., 2011). Microscopically, CTE typically features tau-positive NFTs and astrocytic tangles, and neuropil neurites in a distribution distinctly different from other tauopathies such as Progressive Supranuclear Palsy, Corticobasal degeneration, and Alzheimers' Disease (AD) (McKee et al., 2009; Omalu et al., 2011). In CTE, these changes are patchy, localized to the depths of sulci, perivascularly around small vessels, in subpial areas and in the superficial cortical layers (II/III) (McKee et al., 2009; Omalu et al., 2011). In contrast to AD, CTE, as defined in recent literature (McKee et al., 2009; Omalu et al., 2011) lacks significant amounts of Alzheimer's-like beta-amyloid plaques (McKee et al., 2009; Gavett et al., 2011; Omalu et al., 2011; Stern et al., 2011). The neuropathology of CTE has been recently expanded to encompass in some cases the possible presence of widespread TDP-43-positive inclusions in the brainstem, basal ganglia, and cortex (McKee et al., 2010). In such cases, the presence of these lesions in the corticospinal tract and anterior horn of the spinal cord may be associated with clinical findings of spasticity, weakness, and fasciculations, similar to the clinical presentation ALS (McKee et al., 2010).

Here, we report on the clinical and pathological case histories for 6 retired Canadian Football League (CFL) athletes who underwent autopsies limited to the central nervous system. We address the question whether retired professional athletes with a history of multiple concussions and the presence of neurological findings will invariably manifest as CTE (alone or with co-morbid pathology) or whether this history can be associated with other diagnoses.

## Methods

This study was approved by the Ethics Review Board of the University Health Network. Informed consent to participate in this study was provided by each participant or the participant's designated next of kin.

### Participants

The brains of six adults consecutively referred for autopsy were examined. Participants comprised a convenience sample of adults who played professional football (CFL) with a history of multiple concussions and medically- or family-member documented histories of progressive cognitive, psychiatric, and/or motor symptoms (see Table 1).

**Table 1:** **Summary of clinical histories of professional football athletes in study cohort**.

**Case**	**Age at onset (yrs)**	**Initial symptoms**	**Progressive dementia**	**Behavioral changes**	**Language**	**Memory decline**	**Executive function**	**VSP**	**Motor impairment**	**Disease duration (yrs)**	**Concussions**	**Family history**	**Pathological diagnosis**
1	70	Apathy, Memory deficits, decreased concentration, getting lost, language deficits	Yes	Apathy; rummaging through garbage, irritable, aggressive late	Severe expressive aphasia (nonfluent) 2005	STM	Decreased concentration	Getting lost	Very late (2010)	16	Multiple	Brother (late onset AD), Paternal aunt and Grandfather (dementia). Parents died young.	1. CTE
													2. AD (Braak VI/VI)
2	56	Pseudobulbar affect	No	No	Mild word- finding deficits late	Mild STM (late onset)	Mild difficulty with planning/organizing, problem-solving very late	No	Dysarthria/dysphagia	5	Multiple	Nil	1. ALS
3	50	Personality- affect flatter; appeared depressed; STM; subtle changes in gait	Yes	1994 MDE, apathy, less empathy, dis-inhibited, agitation/aggression, anxious, paranoid delusions, hallucinations	Later moderate expression/comprehension deficits	STM	Decreased concentration late 90s	No	Slower gait, Instability, Tremor, Rigidity, Parkinsonian Gait and RBD, Lost sense of olfaction.	29	Multiple	Sister (PD) Mother & Father (depression)	1. DLBD
													2. CTE
4	55	Memory deficits, apathetic, depressed	Yes	Apathetic, agitated, depressed Later paranoid delusions	Word-finding initially then loss of semantic meaning, word substitution	STM	Financial trouble 2009 because of poor judgment	Getting lost	Difficulty walking because of toe amputation	12	Multiple	Vascular dementia paternal grandfather	1. CTE
													2. Multiple infarcts
5	64	Memory deficits, irritability	Yes	Aggressive, apathetic, hallucinations, delusions	Word-finding difficulty, increased speech output	STM	Poor judgment early on–let people leave with articles without paying	Getting lost	No	10	Multiple	Mother late onset AD, Father late onset dementia	1. AD
6	48	Motor-slowing; anxious, withdrawn	Yes	Hallucinations; delusions; throwing everything away	Decreased speech output	STM	Loss of judgment, loss of planning/organizing	Getting lost (late onset)	Dysphagia, Dysarthria, Bradykinesia	15	Multiple	Nil	1. PD

Abbreviations: MDE, major depressive episode; STM, short-term memory; VSP, visuospatial function; PD, Parkinson's disease; AD, Alzheimer's disease; ALS, Amyotrophic Lateral Sclerosis; DLBD, Diffuse Lewy body disease. “Disease Duration” indicates number of years between initial symptoms and death.

### Design

This was a retrospective, case series design of consecutively referred brains for autopsy. Analyses are descriptive, using the following possible primary outcomes: neuropathological diagnosis of (i) CTE alone, (ii) CTE plus other neurological disorder, (iii) other neurodegenerative disorder and (iv) no neurodegenerative disorder.

### Procedures

Clinical details were collected from next of kin, treating physicians, and medical records. The clinical data was obtained through structured interviews of the family members as well through clinical consult notes sent by treating physicians. The brain autopsy was authorized by next of kin.

#### Neuropathological analysis protocol

The post-mortem time varied from 4 to 72 h. At autopsy, the brains were immediately placed in neutral formalin and sectioned after two weeks. At the time of autopsy, a piece of frontal lobe was snap frozen for future proteomic/genetic studies. The brains were photographed and extensively sampled from several cortical, subcortical, cerebellar and brainstem areas. Tissue blocks were processed and embedded in paraffin. Six micron coronal sections were stained with Luxol fast blue and hematoxylin and eosin (H&E/LFB), Bielschowsky silver impregnation or Gallyas silver stain, and by immunohistochemistry with the following antibodies: hyperphosphorylated-tau (mouse monoclonal AT8; Pierce Endogen, Rockford IL; 1:2000), [alpha]-synuclein (rabbit polyclonal; Chemicon, Temecula, CA; 1:15,000), and A[beta] (mouse monoclonal, Dako North America Inc., Carpinteria, CA; 1:2000) (after formic acid pretreatment). Other antibodies used for immunostaining were: glial fibrillary acidic protein (GFAP-Chemicon; 1:2000), TDP-43 (rabbit polyclonal to TAR DNA-binding protein, 1:1000; Abcam, Cambridge, MA), and ubiquitin (rabbit polyclonal, 1:2000; Dako North America Inc., Carpinteria, CA). The neuropathological diagnoses were based on most recent neuropathological criteria provided by consensus studies for AD (Hyman et al., 2012), Parkinson's disease (PD) (Dickson, 2012) and fronto-temporal dementia (Cairns et al., 2007). CTE diagnosis was based on recent publications (McKee et al., 2009; Omalu et al., 2011).

## Results

### Overview of cases

All six participants had been professional football athletes in the CFL, and had played multiple positions including offensive and defensive positions. All participants had histories of multiple concussions, but information about the exact frequency and intensity of head injury could not be determined. Their ages ranged from 61 to 87 years with disease durations of 3–17 years from the onset of first reported neurodegenerative symptoms to death. Additional details are provided in Table 1.

### Case findings

#### Case 1

***Sport history***. This patient played football from a young age, continued through university and played professionally in the CFL for 5 years. He played multiple positions, but was predominantly a halfback and kick returner. He had many concussions.

***Clinical history***. Around age 70, the patient first developed memory impairment, including getting lost, and difficulty concentrating. Apathy was also noted in his early 70s. His hygiene was not impaired, but he rummaged through the family's garbage and pulled out old items. His eating habits changed, as he became bothered by the texture of certain foods, especially meat. He was a restless sleeper, but did not display violent behavior during sleep. He also developed language deficits including word-finding difficulty and semantic paraphasias. By 85 he had developed significant expressive aphasia while his comprehension remained intact until a few weeks before his death. He also showed significant neuropsychiatric symptoms that included increased irritability and agitation. His motor function was preserved until late in his course of illness; he was still walking until age 86 when he became confined to a wheelchair due to unsteadiness. The patient died at age 86 years.

***Family history***. His family history was significant for a brother who developed AD in his 70's and dementia in his paternal aunt and grandfather. Both his parents died young from unrelated causes.

**Figure 1:**
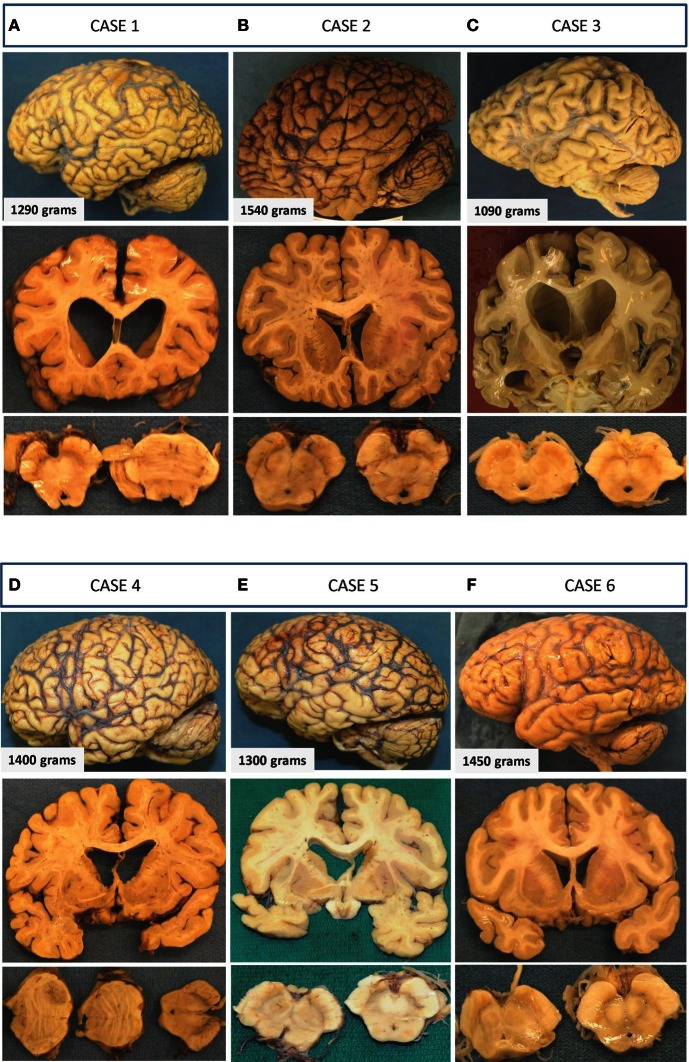
**Gross macroscopic findings on neuropathological examination.** Selected examples from each case are presented as three vertical panels with lateral views of the unsectioned brain (upper panel), coronal sections through various parts of the cerebrum (middle panel), and axial sections through the brainstem (lower panel). **(A) Case 1:** There is mild-to-moderate atrophy with ventricular enlargement and cavum septi pellucidi. Thinning of the corpus callosum and mild depigmentation of the substantia nigra is also evident. **(B) Case 2:** Unsectioned brain, ventricles, and sections of the midbrain and substantia nigra appear within normal limits with no apparent evidence of neurodegeneration. **(C) Case 3**: Preferential mild-to-moderate atrophy of frontal and temporal lobes with significant enlargement of ventricles seen on coronal sections. A fenestrated septum pellucidum and atrophied amygdala and hippocampus are also seen on this section. The substantia nigra of this patient shows significant loss of pigmentation. **(D) Case 4:** There is mild frontal and temporal lobe atrophy with enlarged ventricles and cavum septi pellucidi. Axial sections of the brainstem show normal appearing substantia nigra and a metastatic lesion in the pons. **(E) Case 5:** Atrophic brain is seen with moderately enlarged ventricles and a normally pigmented substantia nigra. **(F) Case 6:** Minimal atrophy and ventricular enlargement are seen, and there is mild loss in the substantia nigra.

***Neuropathological findings***. Neuropathological examination showed a moderately atrophic brain (1290 g) with mild but preferential wasting of the frontal and temporal lobes (Figure 1). The ventricles (including the temporal horns of the lateral ventricles) were moderately enlarged. There was cavum septi pellucidi, thinning of the corpus callosum and atrophy of the amygdala and mamillary bodies. The hippocampi appeared normal in size. Examination of the midbrain revealed depigmentation of the substantia nigra (SN) (Figure 1).

**Figure 2:**
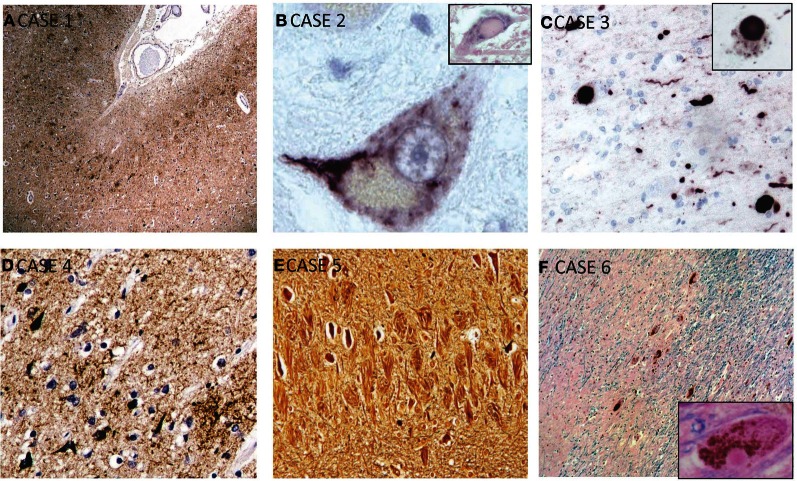
**Selected microscopic findings on neuropathological examination. (A) Case 1:** High power view of sample of cerebral cortex stained for hyperphosphorylated-tau shows concentration of neurofibrillary tangles predominantly in the superficial layers of the gray matter and in the depths of sulci, which is characteristic of CTE. **(B) Case 2:** Magnified motor neuron in ventral horn of spinal cord shows a representative intracytoplasmic TDP-43 inclusion. Inset shows a hyalin inclusion. **(C) Case 3:** Alpha-synuclein staining showing Lewy bodies and neurites which are seen throughout the cortex, substantia nigra, and locus ceruleus. This patient also had features of CTE (not shown). Inset shows higher power view of cytoplasmic Lewy body. **(D) Case 4:** Immunohistochemisty showing widespread reactivity against tau-positive neurofibrillary and astrocytic tangles in multiple layers. **(E) Case 5:** Bielschowsky silver stain showing numerous neurofibrillary tombstones in the hippocampus. **(F) Case 6:** HandE/LFB and alpha-synuclein staining of representative cortical section showing diffuse Lewy bodies and Lewy neurites. Inset shows Lewy body.

Microscopically, the brain showed widespread tauopathy. More specifically, hyperphosphorylated-tau staining showed concentration of neurofibrillary tangles (NFTs) predominantly in the superficial layers in the gray matter and depths of sulci (Figure 2). There was continuous tau-positive glia in the subpial and patchy areas seen in the grey/white matter junction and around blood vessels. Numerous NFT were also noted in the deeper layers of the cortex as well. Tau distribution was diffuse throughout the brain involving the frontal, temporal (highest populations), and inferior parietal lobes, indusium griseum, and striate and cingulate cortices. There was heavy tau staining in the amygdala and throughout the hippocampus (CA1-4, subiculum and trans-enthorhinal cortex). There were patchy tau-positive inclusions seen throughout the brainstem, as well as in the nucleus basalis of Meynert, the thalamus, hypothalamus, and mammillary bodies. Numerous senile plaques were observed throughout the brain, most notably in the trans-entorhinal cortex. TDP-43 staining was not a feature of this case.

***Neuropathological diagnosis***. Overall, the pathological patterns of staining showed characteristics of both CTE and severe AD (Braak Stage VI/VI).

#### Case 2

***Sport history***. This participant played football, hockey, and rugby from a young age, including 12 years in the CFL playing defense. He had many concussions.

***Clinical history***. The patient first developed emotional lability and slurred speech at age 56 years, and was subsequently diagnosed with bulbar onset ALS. Over the next few years, he lost his speech and ability to swallow, requiring a feeding tube at age 59 and a tracheotomy at age 61. His limb movements, personality, and cognitive function remained relatively preserved, although there was a mild decline in short memory function at age 60. He died at age 61.

***Family history***. No family history of a neurodegenerative disease.

***Neuropathological findings***. Neuropathological examination showed normal brain weight (1540 g) and normal exterior appearance without atrophy (Figure 1). Ventricles were of normal size with no cavum septi pellucid. SN showed normal pigmentation.

Microscopic examination revealed loss of neurons in the motor nuclei of multiple cranial nerves—predominantly cranial nerves VII and XII—with TDP-43 positive intracytoplasmic inclusions (Figure 2). Some intracytoplasmic TDP-43-positive inclusions and neuronal loss was also noted in the cervical spinal cord involving the lower motor neurons. Inclusions were also noted in the primary motor cortex, and to a lesser extent, in the dentate gyrus. Pathological deposition of hyperphosphorylated tau was very scarce and limited to the trans-entorhinal cortex in the shape of NFTs in neurons. A few beta-amyloid plaques were also noted.

***Neuropathological diagnosis***. These neuropathological findings were consistent with the diagnosis of amyotrophic lateral sclerosis (ALS).

#### Case 3

***Sport history***. This participant retired after a12-year CFL career, playing both offense and defense positions. He had many concussions.

***Clinical findings***. His wife noticed personality changes, including flat affect and depressed mood, when he was age 50. At age 55, he developed some short-term memory impairment. In his 60s, his family noticed executive deficits in that he could no longer manage his company. At the age of 62, she noticed some subtle changes in his gait. He walked more slowly, and less steadily. He also had a major depressive episode that year and underwent electroconvulsive therapy. He developed a tremor, rigidity, a parkinsonian gait, and rapid eye movement (REM) sleep behavior disorder, and also lost his sense of olfaction. His wife also noted apathy and reduced empathy. In his early 70s, he became disinhibited, had delusions, hallucinations, and episodes of agitation and aggression. He was diagnosed with PD, but the early cognitive changes at the onset of his disease and the subsequent hallucinations were more typical of dementia with Lewy bodies (DLB). The patient died at age 79.

***Family history***. His sister was reported to have PD. Both his parents suffered from depression.

***Neuropathological findings***. Neuropathological examination of the brain revealed a moderately atrophic brain with a weight of 1090 g after fixation (Figure 1). Moderate volume loss was noted in the frontal, temporal, and parietal lobes, and mild atrophy was noted in the occipital lobe. There was significant ventricular enlargement, thinning of the corpus callosum, and cavum septi pellucidi. Coronal sectioning of the brain revealed significant atrophy of the amygdala and hippocampus. The brainstem examination revealed pallor of the SN (Figure 1).

Microscopic examination revealed a widespread tauopathy in the form of neurofibrillary and astrocytic tangles clustering in patches in the superficial layers of most cortical areas in both the sulci and gyral crowns. The primary visual cortex was spared. Tau-immunopositive neurons were most pronounced in the amygdala and hippocampus. There were diffuse astrocytic tangles noted around blood vessels and throughout the parenchyma. Tau-positive inclusions and neurites also populated the subcortical structures including the striatum, globus pallidus, dentate nucleus of the cerebellum, thalamus, subthalamic nucleus, substantia nigra, hypothalamus, septal nuclei, nucleus basalis of Meynert, mammillary bodies, periventricular white matter, locus ceruleus, red nucleus and the nucleus of the third cranial nerve. Localized TDP-43 staining of the amygdala and hippocampus revealed numerous inclusions. Alpha-synuclein staining revealed numerous Lewy bodies and Lewy neurites throughout the cortex, substantia nigra and locus ceruleus suggested advanced Lewy body disease (Figure 2). There were senile plaques in the hippocampus and cortical areas that were tau-positive, and stained with Congo red and Gallyas silver. Luxol fast blue stain showed abnormal pallor of the white matter. There was also neuronal loss in the substantia nigra, locus ceruleus, hippocampus, and nucleus basalis of Meynert. Given the extensive neuronal and astrocytic tauopathy, TDP-43 inclusions and synucleinopathy.

***Neuropathological diagnosis***. This case was diagnosed with combined CTE and diffuse Lewy body disease.

#### Case 4

***Sport history***. This participant played football in high school and in the CFL for 6 years. He had many concussions.

***Clinical findings***. When the patient was 55-years old, his sister noted that he had difficulty with short-term memory and that he was less able to formulate arguments. He subsequently developed visuospatial impairments and was reported to get lost in familiar environments. He developed apathy and agitation, and became very depressed. His loss of judgment led to bankruptcy and at the age of 66, he became a ward of the state. He developed language deficits and exhibited word substitution, and was incoherent at times. He became depressed and had paranoid delusions. His past medical history was unremarkable except for recurrent phlebitis. The patient developed lung cancer and died of its complications at age 67.

***Family history***. His paternal grandfather had vascular dementia.

***Neuropathological findings***. Neuropathological examination revealed a brain weight of 1400 g with mild atrophy of the frontal and temporal lobes (Figure 1). There were findings consistent with widespread metastatic disease from a lung carcinoma and severe vascular atherosclerotic disease with recent multifocal brain infarctions. There was also thinning of the olfactory tracts and hypothalamus. Coronal sections of the brain showed an enlarged ventricular system, corpus callosum atrophy, and cavum septi pellucidi. Pigmentation of the SN appeared within normal limits (Figure 1).

Microscopically, there was mild to moderate neuronal loss and gliosis in CA1, subiculum, entorhinal cortex, amygdala, mammillary bodies, and medial thalamic nuclei. There was granulovacuolar degeneration noted in the CA1 and subiculum area with pronounced subpial gliosis in the trans-entorhinal cortex. Immunohistochemically, there was widespread tau-positive neurofibrillary and astrocytic tangles in multiple layers (superficial > deep) of the cortex, especially in the depths of sulci (Figure 2). There were some inclusions noted in the gyral crowns. These inclusions were consistently found in all cortical areas with a predilection for the medial temporal, hippocampus, and amygdala areas. NFTs were also noted in thalamus, peri-ventricular hypothalamic areas extending into the mamillary bodies, the nucleus basalis of Meynert and clustering around blood vessels. Beta-amyloid staining revealed amyloid plaques. TDP-43 and alpha-synuclein staining were unremarkable.

***Neuropathological diagnosis***. Overall, neuropathological findings and the distribution of tauopathy were compatible with changes seen in CTE. Although there were multiple infarcts noted, they appeared to be relatively recent infarcts and could not fully account for the participant's longer-term dementia.

#### Case 5

***Sport history***. This patient played in the CFL for 8 years. He had many concussions.

***Clinical findings***. At age 64, he began to show behavioral changes, including anger, poor judgment, and irritability. Over the next few years his memory worsened and he began to get lost. He then began having hallucinations of strangers in his home and also developed some misidentification for others and himself. He seemed to no longer recognize himself as he would attack mirrors when he walked in front of them. He developed delusions that people were stealing from him and had episodes of aggression and agitation, as well as impaired motor function. The patient died at age 74.

***Family history***. His mother had late onset AD and his father had late onset dementia.

***Neuropathological findings***. His brain was atrophic with a weight of 1300 g after fixation with moderate ventricular enlargement (Figure 1). SN showed normal pigmentation.

Microscopic examination revealed numerous NFTs in neurons of the deep cortical layers (Figure 2). These were concentrated to the trans-entorhinal cortex, hippocampus, and isocortex with significant extension into the primary visual cortex. There was also significant presence of tangles in nucleus basalis of Meynert, amygdala, substantia nigra, and in the Edinger–Westphal nucleus. Supplementing the tangles were numerous dense-core, beta-amyloid positive plaques. No evident TDP-43 or alpha-synuclein staining was seen.

***Neuropathological diagnosis***. Overall, findings were consistent with severe AD (Braak Stage VI/VI) without any pathological evidence of CTE.

#### Case 6

***Sport history***. He began playing football in high school and played seven years in the CFL. He suffered multiple concussions.

***Clinical findings***. At age 48, his wife noted that he was becoming withdrawn and then he gradually changed from a confident, assertive, energetic person to an anxious, insecure, and more lethargic person. At age 50, he noted his handwriting had become messier, and also complained of some cramping and numbness in his feet and decreased ability for playing baseball. He also began exhibiting memory deficits, which became progressively worse over the subsequent years. A few years later, his wife noted his speech was slurred and hypophonic and the slowing of his movements became more apparent. He did not have a tremor. At age 54, his wife became concerned about his judgment based on poor business decisions. He became obsessive about bladder incontinence and went to the bathroom multiple times a day, yet seemed incongruously unperturbed when accidents did happen. By age 55, he was having repeated episodes of loss of bladder control. He had vivid dreams that he was convinced were real, but no REM sleep behavior disorder. His judgment continued to deteriorate, and he became less attentive to hygiene. He had delusions and hallucinations, but these stopped with discontinuation of Sinemet therapy. He eventually had episodes of agitation and developed great difficulty ambulating. He had prosopagnosia at age 58. At that time, he began showing difficulty recalling the names of his children. His past medical history was unremarkable. The patient died at age 63.

***Family history***. No family history of a neurodegenerative disease.

***Neuropathological findings***. Neuropathological examination showed a brain of normal weight (1450 g) with mild diffuse cortical atrophy. Ventricles were mildly dilated (Figure 1).

On microscopic examination, there was diffuse Lewy body disease with Lewy bodies and Lewy neuritis in the cerebral cortex, olfactory bulbs, indusium griseum, SN and limbic system, including the CA2-4 subdivisions of the hippocampus (Figure 2). There was extensive neuronal loss in the SN pars compacta, locus ceruleus, dorsal nucleus of CN X and nucleus basalis of Meynert. There was very limited tau labeling in the hippocampus, the amygdala, and peri-amygdala cortex. There was, however, widespread distribution of diffuse amyloid plaques. TDP-43 staining in this case was unremarkable.

***Neuropathological diagnosis***. Overall, this case showed typical changes of long-term progressive PD. While the tau-deposits in the hippocampal area were age-appropriate Alzheimer-type changes (Braak Stage II/VI), there was no evidence of AD. There was also no pathological evidence consistent with CTE.

## Discussion

At present, the diagnosis of CTE requires post-mortem examination. In our case series, a history of participation in professional football and a history of multiple concussions, combined with positive clinical signs and symptoms of progressive neurodegenerative disease, were not inevitably associated in each of the 6 cases with a post-mortem diagnosis of CTE. The neuropathological diagnosis of our six cases comprised: CTE + AD, CTE + diffuse Lewy body disease, CTE + multiple infarcts, AD, ALS, and PD (see Table 1). In our case series of professional football athletes, we observed that the reported progressive neurological findings in some athletes participating in contact sports were associated with CTE, while other athletes had more common neurodegenerative conditions, namely AD, PD, and ALS. Moreover, those individuals with post-mortem diagnosis of CTE had co-morbid pathological findings that may also have contributed to the clinical signs and symptoms. Thus, our findings advocate caution in the clinical diagnosis of CTE in patients with histories of contact sports and neurocognitive decline, as other diagnoses of neurodegenerative diseases are also possible. Our findings are consistent with a literature review by Nowak et al. (2009), in which dementia in retired boxers could be explained by pathologies aside from dementia pugilistica (Nowak et al., 2009—see also McKee et al., 2013). In contrast, other previous studies either focused on describing CTE in professional athletes (Omalu et al., 2005, 2006, 2010b,c; McKee et al., 2009, 2010) or found that a majority of professional athletes had CTE (Omalu et al., 2011).

These findings raise questions regarding the relationship between multiple concussions in professional football alumni and CTE, the prevalence of CTE in this population and the risk factors. Previous post-mortem research with larger samples of professional athletes with multiple concussions has suggested a very high incidence rate; however, such studies have been limited by biased samples restricted to clinically symptomatic cases and a lack of medical post-mortem controls with co-morbidities consistent with the professional athlete histories, including comparable medication/substance histories as well as pain disorders, of potential relevance given increasing evidence for the role of neuroinflammatory processes in pain disorders (Davis and Moayedi, 2012).

Our findings cannot address these limitations, but suggest the testable hypothesis that the mapping between multiple concussion history in former professional athletes plus positive progressive clinical findings is not one-to-one with CTE, a hypothesis supported by the expectation that aging professional athletes should be as susceptible as the general aging population to neurodegenerative diseases such as AD, vascular dementia, PD or ALS.

A limitation of our retrospective clinical case series was that historical information was subject to recall bias. The participants' informants could not provide the actual number or severity of the concussions, although there was sufficient information to indicate that each player had sustained multiple concussions throughout their careers. When these players were active participants, concussions were not well recognized, and if recognized, were treated as a minor injury.

## Conclusion

Our initial experience with this cohort of retired professional football athletes with multiple concussions and progressive neurocognitive decline demonstrates that these cases did not uniformly have neuropathological findings of CTE. Some cases with CTE pathology had concomitant pathologies that could also contribute to cognitive decline. Thus, it is difficult to establish a definitive link between a history of multiple concussions and CTE. Neuropathological examination remains essential for diagnosis of CTE, as other types of brain degeneration may be present in professional athletes with neurocognitive decline. Further research is needed to establish the relationship between multiple concussions and the development of CTE and to examine the prevalence and the risk factors that mediate the relationship between multiple concussions and development of CTE.

### Conflict of interest statement

The authors declare that the research was conducted in the absence of any commercial or financial relationships that could be construed as a potential conflict of interest.
